# Low prevalence of sub-microscopic malaria among rural communities of an endemic area in Kapit Division, Malaysian Borneo

**DOI:** 10.1186/s12936-026-06057-x

**Published:** 2026-07-12

**Authors:** Siti Farah Norasyikeen Sidi Omar, Romano Ngui, Sam Froze Jiee, Siong Hee Lim, Timothy Jinam, Melvin Hsien Liang Chung, Micheal Pelitini Ugak, Drend Jores, Paul Cliff Simon Divis, Yvonne Ai Lian Lim

**Affiliations:** 1 Department of Paraclinical Sciences, Faculty of Medicine and Health Sciences, Universiti Malaysia Sarawak, 94300 Kota Samarahan, Sarawak Malaysia; 2 Department of Community Medicine and Public Health, Faculty of Medicine and Health Sciences, Universiti Malaysia Sarawak, 94300 Kota Samarahan, Sarawak Malaysia; 3 Bintulu Division of Health Office, Jalan Tun Ahmad Zaidi, 97000 Bintulu, Sarawak Malaysia; 4 Kapit Division of Health Office, Tingkat 3, Wisma Persekutuan, 96800 Kapit, Sarawak Malaysia; 5 Sarikei Division of Health Office, Tingkat 3, Wisma Persekutuan, 96100 Sarikei, Sarawak Malaysia; 6 Malaria Research Center, Faculty of Medicine and Health Sciences, Universiti Malaysia Sarawak, 94300 Kota Samarahan, Sarawak Malaysia; 7 Department of Parasitology, Faculty of Medicine, Universiti Malaya, 50603 Kuala Lumpur, Malaysia

**Keywords:** Malaria, Submicroscopic malaria, Kapit Division, Sarawak, Malaysia

## Abstract

**Background:**

Malaria remains a major public health concern, especially with zoonotic *Plasmodium knowlesi* presenting specific challenges in Southeast Asia. In Malaysia, Sarawak ranks second in *P. knowlesi* cases after Sabah. Rural populations in endemic areas experience increased exposure to forest and forest-edge zones where vector mosquitoes breed, thereby raising infection risk and complicating routine surveillance. These groups may carry asymptomatic or submicroscopic infections that act as hidden reservoirs, potentially hindering elimination efforts. This study aimed to assess the prevalence of *Plasmodium* infections, including submicroscopic cases, among rural communities in Kapit Division, a malaria-endemic area.

**Methods:**

A cross-sectional study was conducted from March 2024 to August 2025, including 376 participants aged 2 to 90 years. Venous blood samples were examined under a light microscope with Giemsa-stained thick and thin smears. Additionally, all samples underwent species-specific nested PCR to verify microscopy results and identify sub-microscopic infections. Hemoglobin levels were measured using a HemoCue® Hb 201 + analyzer. Face-to-face interviews were conducted using a standardized questionnaire. The data analysis was performed with IBM SPSS version 27.0.

**Results:**

The overall malaria prevalence was 5.32% (20/376; 95% CI 3.47–8.07%). Of these, 18 (90%; 18/20) infected individuals were febrile and microscopy-positive. Nested PCR detected two additional afebrile, submicroscopic infections (10%; 2/20). Molecular speciation identified 16 *P. knowlesi*, one *P. falciparum*, and one *P. vivax* among febrile participants. Both submicroscopic cases were *P. knowlesi*. All infections occurred in the Kapit District. Anemia was found in 17.6% (66/376) of participants. Of these, 4 were positive for *P. knowlesi*, with no significant association with the infection. Infection was significantly associated with age over 40 years (p = 0.02), non-field employment (p = 0.003), and higher household income (p < 0.001).

**Conclusion:**

This study shows low *Plasmodium* infection rates in Kapit, with some submicroscopic infections. This emphasizes the importance of molecular surveillance in eliminating malaria, as traditional diagnostics may overlook hidden reservoirs. To monitor ongoing transmission, prevent re-establishment of local human malaria, and control zoonotic *P. knowlesi*, routine surveillance should include both PCR and microscopy in endemic areas.

## Background

Malaria continues to pose a formidable global public health challenge, with an estimated 282 million cases and 610,000 deaths reported in 2024 [1]. In response, the World Health Organization (WHO) launched the Global Technical Strategy for Malaria 2016–2030, aiming for a malaria-free world by 2030. A key milestone toward this goal requires countries to achieve three consecutive years of zero indigenous transmission before being certified malaria-free [2]. Malaysia made remarkable progress toward elimination, notably recording zero indigenous cases of human malaria (i.e., *Plasmodium falciparum*, *Plasmodium vivax*, and *Plasmodium malariae*) in 2018 through its Malaria Eradication Program [1].

However, this achievement has been challenged by the persistent and rising incidence of zoonotic malaria, primarily caused by *Plasmodium knowlesi*. Environmental changes, such as deforestation and agricultural expansion, have increased human encroachment into forested areas, thereby elevating contact between humans, macaque reservoirs, and competent mosquito vectors [3]. Between 2018 and 2022, *P. knowlesi* remained the sole cause of indigenous malaria transmission in Malaysia [4], with annual case numbers fluctuating between 2000 and 3000 [1]. According to the WHO Malaria Report 2025, *P. knowlesi* now makes up 89% of all reported cases in Malaysia [1]*.* This pattern is particularly evident in Malaysian Borneo states such as Sarawak, where extensive forests and local occupations support continuous zoonotic transmission [5].

A critical complication in elimination efforts is the presence of sub-microscopic and asymptomatic malaria infections. These low-parasitemia infections frequently evade detection by routine light microscopy and require more sensitive molecular diagnostics, such as PCR [6]. Asymptomatic individuals do not seek treatment, allowing parasites to persist undetected. While asymptomatic and submicroscopic infections have been implicated in sustaining transmission of human malaria species, their contribution to *P. knowlesi* transmission remains incompletely understood [6]. This challenge is particularly pronounced in rural communities, such as those in Sarawak, where livelihoods often depend on activities near forests, including farming, hunting, and logging [7–9]. In these settings, housing conditions often provide limited protection against vectors, and healthcare access is restricted, further establishing these communities as potential reservoirs for ongoing transmission [10].

Together, these factors indicate that traditional surveillance methods might underestimate the true burden of malaria in endemic regions. While asymptomatic and sub-microscopic infections are increasingly reported in Peninsular Malaysia [6, 11–17], information from rural communities in Sarawak, particularly in endemic areas, remains limited and poorly understood [18, 19]. This surveillance gap hinders evidence-based interventions and risks undermining Malaysia’s progress toward elimination. Therefore, this study aimed to determine the prevalence of *Plasmodium* infections, including submicroscopic cases, among rural communities in the Kapit Division. The findings are expected to provide contemporary insights into the local malaria burden and inform targeted surveillance and control strategies to support Malaysia’s malaria elimination goals.

## Methods

### Study design and areas

A cross-sectional study was conducted in the Kapit Division, the largest administrative division in Sarawak, Malaysia. Kapit Division is further divided into three districts: Kapit, Song, and Belaga. The division covers approximately 38,934 km^2^ and had an estimated population of 134,800 in the 2020 national census. The region has become increasingly accessible following the recent completion of road infrastructure connecting Kapit with Sibu, the nearest major urban center. According to official health surveillance data, Kapit Division is classified as an endemic area for malaria, with *P. knowlesi* as the predominant species [20].

**Fig. 1:**
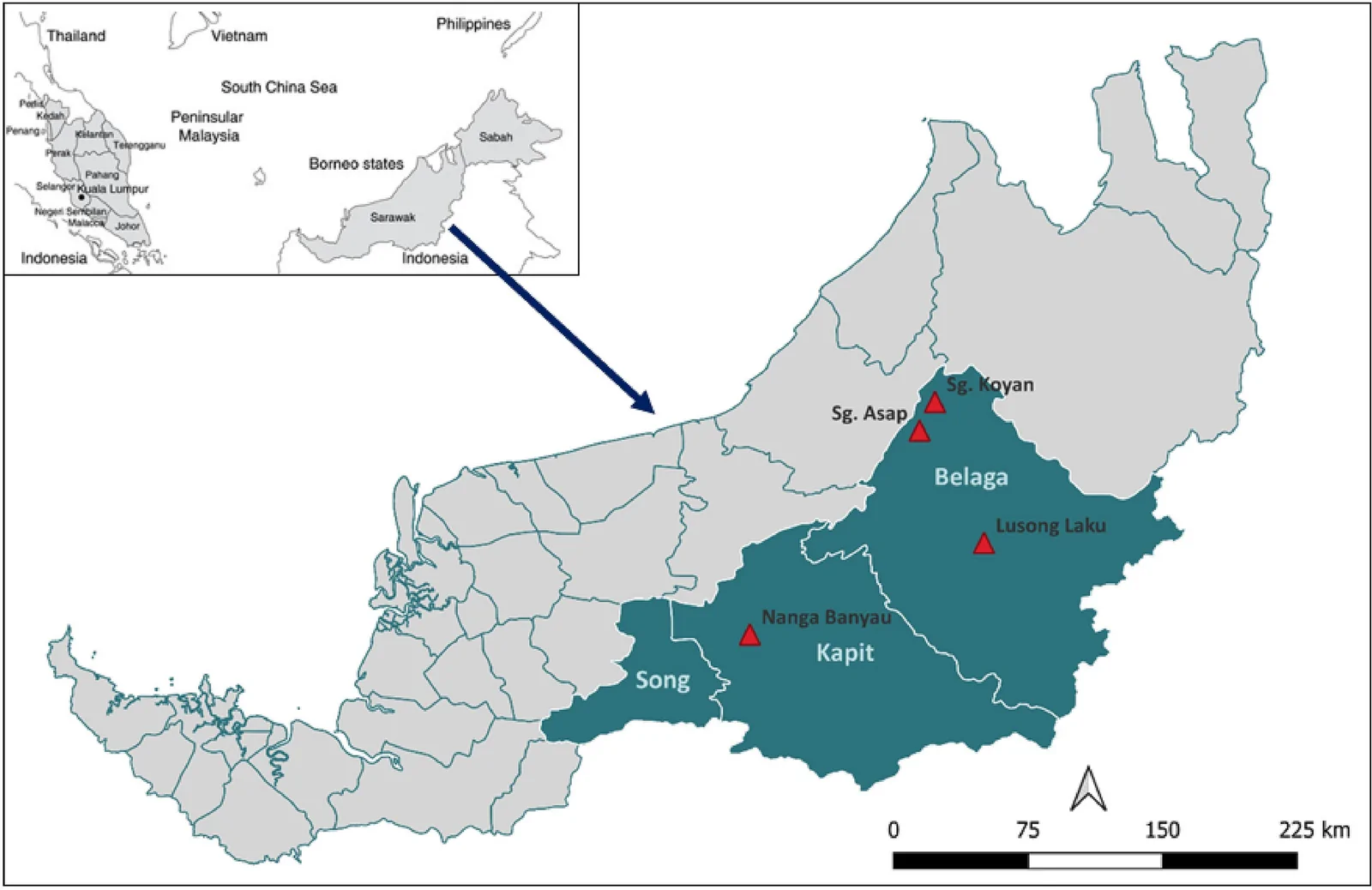
Map of Kapit Division in Borneo, Sarawak, showing the study area

The study sites comprised traditional longhouses in rural areas across the Kapit Division. Longhouses are communal dwellings that house multiple families or entire villages and are characteristic of indigenous communities in Sarawak. Using a convenience sampling approach, longhouses were selected based on recommendations from the Kapit Divisional Health Office and accessibility via the road network. Sampling sites were situated in two districts: Kapit District (i.e., Nanga Banyau) and Belaga District (i.e., Lusong Laku, Sungai Asap, and Sungai Koyan). These areas are predominantly located near forest fringes and along the banks of major rivers, including the Rajang and Baleh, which influence local vector ecology and human movement patterns. The geographic distribution of the study sites is shown in Fig. 1.

### Sample size determination

The minimum sample size was calculated using the single population proportion formula [21]. Based on the recent reported submicroscopic malaria prevalence of 15.3% among indigenous populations in Malaysia [11], and assuming a 95% confidence level and a 5% margin of error, a minimum of 199 participants were required. To account for potential nonresponse or incomplete data, the sample size was increased by 20%, resulting in a final target of 239 participants. Participants were eligible if they were aged 1 year or older, regardless of gender, were registered residents of Kapit Division, had no recent travel history outside the study area, and had not received antimalarial treatment in the 4 weeks preceding enrolment. Individuals who declined informed consent or those currently under treatment for malaria were excluded from the study. In addition, pregnant women were excluded from this community-based surveillance to ensure a consistent non-pregnant reference population for our prevalence, hematological analyses, and related factors in the general population.

### Sociodemographic data collection

A pretested questionnaire was administered face-to-face to the head of each participating household, who served as the representative respondent. The questionnaire was provided in both English and Bahasa Malaysia. The Bahasa Malaysia version, a national language of Malaysia, was used to collect sociodemographic, socioeconomic, and clinical information. Variables included age, gender, ethnicity, education level, occupation, household income, recent history of malaria or febrile illness, and self-reported symptoms at or before sample collection.

### Blood collection

Approximately 5 mL of peripheral venous blood was drawn by trained nurses or medical assistants into EDTA-anticoagulated vacuum tubes. A small aliquot of fresh blood was immediately used to measure hemoglobin levels to screen for anemia. EDTA samples were placed in a cooler box with ice packs and transported to the Parasitology Laboratory, Faculty of Medicine and Health Sciences, UNIMAS, where they were stored at − 20 °C until further analysis.

### Hemoglobin (Hb) measurement

Finger-prick blood samples were obtained from participants to assess Hb levels. Hemoglobin was measured in the field using a portable HemoCue® Hb 201 + analyzer (HemoCue AB, Sweden). The analyzer was calibrated against the international reference method for Hb concentration, in accordance with the manufacturer’s instructions. The cut-off value for anemia was determined according to the WHO guidelines. Anemia was classified according to WHO age- and sex-specific hemoglobin thresholds criteria for children aged 2–14 years, non-pregnant women and men: mild (Hb 10.0–10.9 g/dL [children 6–59 months], 11.0–11.9 g/dL [non-pregnant women/children 5–14 years] or 11.0–12.9 g/dL [men]), moderate (Hb 7.0–9.9 g/dL [children 6–59 months] or 8.0–10.9 g/dL [children aged 5–14 years, non-pregnant women, men]) and for severe anemia (Hb < 7.0 g/dL [children 6–59 months] < 8.0 g/dL [children aged 5–14 years, non-pregnant women, men]) [22].

### Microscopic examination

Both thin and thick smears were prepared immediately after blood collection in the field by a trained medical team. For the thin smear, a small blood drop (3–5 µL) was placed near one end of a slide, spread at 30–45° with a spreader slide to form a thin layer, air-dried, and fixed with methanol for 30 s. The thick smear involved placing a larger drop (10–15 µL) on a slide, spreading it in a circle, and drying for 30 min to lyse red blood cells and concentrate parasites. Both smears were transported to the laboratory for staining. Then, the smears were stained with 3% Giemsa for 30 min, rinsed, air-dried, and examined at 100× oil immersion for malaria parasite identification. Each slide was independently examined by two microscopists, with results validated by an experienced parasitologist. Slides were systematically examined using a zig-zag method. The entire smear was examined before it was deemed positive or negative.

### Molecular detection of *Plasmodium* spp. by nested PCR

Genomic DNA was extracted from EDTA-anticoagulated blood using the PrimeWay Genomic II DNA extraction kit (1st BASE, Malaysia) according to the manufacturer’s protocol. DNA concentration and quality were measured using NanoDrop Spectrophotometers (NanoDrop Technologies, USA), and the extracted DNA was stored at −20 °C until further use. Briefly, the extracted DNA was subjected to nested PCR targeting the 18S ribosomal RNA gene of *Plasmodium* spp. [23]. The first (genus-specific) amplification used PLU1 and rPLU5 primers (nest 1) [24], followed by a nested reaction using rPLU3 and rPLU4 (nest 2) [19] to confirm the presence of *Plasmodium* DNA. Positive sample for *Plasmodium* was further tested with species-specific primers to identify human malaria (i.e., *P. falciparum*, *P. vivax*, *P. malariae*, *Plasmodium ovale*) [23] and simian malaria (i.e., *Plasmodium knowlesi*) [25] using the PCR product of genus-specific nested 1. DNA-free water and DNA from a confirmed malaria-positive sample were included as negative and positive controls, respectively, in each PCR run. Amplification was performed in a Veriti™ Thermal Cycler (Applied Biosystem, USA). The amplified products were visualized on a 2% agarose gel stained with SYBR Safe DNA Gel Stain (Invitrogen™, USA) at 100 V for 30 min.

### Statistical analysis

Data was entered and analyzed using IBM SPSS Statistics for Windows, version 27.0 (IBM Corp., 2021). Data completeness was verified before analysis, and any errors or missing data were excluded from the relevant analyses. Descriptive statistics were used to summarize sociodemographic and clinical characteristics. Bivariate analysis using the Pearson chi-square or Fisher’s exact test was applied where appropriate to assess for associations of *Plasmodium* infections and demographic or sociodemographic factors. Variables showing an association with a p-value < 0.05 were considered statistically significant.

## Results

### Characteristics of the study population

A total of 376 participants were enrolled between March 2024 and August 2025. Demographic characteristics of the study population are presented in Table 1. Participants ranged in age from 2 to 90 years, with a median age of 30 years. The largest age group comprised young adults aged 18–40 years (40.2%), and most participants were female (59.3%). Most (79.5%) were not formally employed, including 41.8% who were housewives, 23.9% who were dependent, and 5.6% who were unemployed men. Formal education was reported by 68.1% of participants, and 81.0% resided in low-income households with a monthly net income below RM 500. Among enrolled participants, 4.8% (18/376) reported a history of malaria infection. At the time of sampling, 4.8% (18/376) presented with self-reported fever, and 17.6% (66/376) had anemia. Among anemic individuals, severity was reported as mild (8.2%; 31/376), moderate (8.2%; 31/376), and severe (1.1%; 4/376), as defined by WHO hemoglobin thresholds [22].

**Table 1:** Demographic characteristics of study participants

Characteristics	Frequency	Proportion (%)
Age (years)		
≤ 17	91	24.2
18–40	151	40.2
> 40	126	33.5
Not stated	8	2.1
Gender		
Female	223	59.3
Male	151	40.2
Not stated	2	0.5
Localities		
Lusung Laku	196	52.1
Sg. Koyan	82	21.8
Nanga Banyau	81	21.5
Sg. Asap	17	4.5
Occupation		
Housewife	157	41.8
Dependent (e.g., Tertiary student, school-age children/minors)	90	23.9
Field workers (e.g., agricultural workers, laborers)	65	17.3
Non-field workers (e.g., public sector employees, government workers, self-employed)	31	8.2
Unemployed/Retiree	21	5.6
Not stated	12	3.2
Education		
Receive Formal Education	256	68.1
No Formal Education	102	27.1
Not stated	18	4.8
Income		
< RM500	305	81.1
> RM500	50	13.3
Not stated	21	5.6
Previous history of malaria infection		
Yes	18	4.8
No	358	95.2
Fever		
Afebrile	358	95.2
Febrile	18	4.8
Anemia status		
Non-anemic	243	64.6
Anemic	66	17.6
Not available	67	17.8

RM: Ringgit Malaysia

### Prevalence of malaria infections and their associated factors

Overall, 5.32% (20/376) of participants were positive for *Plasmodium* infection by nested PCR (Table 2). All infections were mono-infections, with *P. knowlesi* as the predominant species (4.78%, 18/376), followed by *P. falciparum* (0.27%, 1/376) and *P. vivax* (0.27%, 1/376). Out of these 20 positive cases, 18 (90%) were febrile and detected by microscopy. Among these febrile cases, 88.9% (16/18) were *P. knowlesi* cases and detectable by microscopy, while 11.1% (2/18) were asymptomatic and submicroscopic, identified only by nested PCR. The remaining two positive cases were febrile *P. falciparum* (5%, 1/20) and *P. vivax* (5%, 1/20). All positive individuals were referred to the Kapit district clinic for treatment.

**Table 2:** Prevalence of malaria infections in rural communities of Kapit Division, Sarawak

Characteristics	No. of individuals	Malaria positive by microscopy	Malaria positive by nested PCR assay			PCR (%)	95% CI	P-value
			*Plasmodium knowlesi*	*Plasmodium falciparum*	*Plasmodium vivax*			
Overall	376	18	18	1	1	5.32	3.47–8.07	–
Age (years)								
≤ 17	91	0	0	0	0	0.00	0.00–4.05	0.02*
18–40	151	9	7	1	1	5.96	3.17–10.94	
> 40	126	9	11	0	0	8.73	4.94–14.96	
Not stated	8	0	0	0	0	0.00	0.00–32.44	
Gender								
Female	223	8	9	0	0	4.04	2.14–7.49	0.34
Male	151	10	8	1	1	6.62	3.64–11.76	
Not stated	2	0	1	0	0	50.00	9.45–90.55	
Localities								
Lusong Laku	196	0	0	0	0	0.00	0.00–1.92	< 0.001*
Sg. Koyan	82	0	0	0	0	0.00	0.00–4.48	
Nanga Banyau	81	18	18	1	1	24.69	16.59–35.08	
Sg. Asap	17	0	0	0	0	0.00	0.00–18.43	
Occupation								
Housewife	157	6	7	0	0	4.46	2.18–8.92	0.003*
Dependent (e.g., Tertiary student, school-age children/minors)	90	1	1	0	0	1.11	0.20–6.03	
Field workers (e.g., agricultural workers, laborers)	67	5	5	0	0	7.46	3.23–16.31	
Non-field workers (e.g., public sector employees, government workers, self-employed)	29	6	4	1	1	20.69	9.85–38.39	
Unemployed/Retiree	21	0	0	0	0	0.00	0.00–15.46	
Non stated	12	0	1	0	0	8.33	1.49–35.39	
Education								
Receive Formal Education	243	12	12	1	0	5.35	3.15–8.94	0.76
No Formal Education	102	6	5	0	1	5.88	2.72–12.24	
Not stated	18	0	1	0	0	5.56	1.00–25.76	
Income								
< RM500	305	11	11	0	0	3.61	2.03–6.34	< 0.001*
> RM500	50	7	7	1	1	18.00	9.77–30.8	
Not stated	21	0	0	0	0	0.00	0.00–15.46	
Previous history of malaria infection								
Yes	18	2	3	0	0	16.67	5.84–39.22	0.63
No	358	16	15	1	1	4.75	2.99–7.47	
Fever								
Afebrile	358	0	2	0	0	0.56	0.15–2.01	< 0.001*
Febrile	18	18	16	1	1	100.00	82.41–100.00	
Anemia status								
Non-anemic	243	10	8	1	1	4.12	2.25–7.41	0.51
Anemic	66	4	4	0	0	6.06	2.38–14.57	
Not available	67	4	6	0	0	8.96	4.17–18.19	

95% CI: 95% Confidence Interval

^*^Statistically significant at P < 0.05

Infection prevalence was significantly associated with age (P = 0.02), with the highest proportion among participants aged ≥ 40 years (11/126; 8.73%), followed by those aged 18–40 years (9/151; 5.96%). No infections were detected in individuals under 18. While prevalence did not differ significantly by gender (P = 0.34), it was marginally higher in males (10/151; 6.62%) than in females (9/223; 4.04%). Geographically, all 20 cases were identified in the Kapit District (i.e., Nanga Bayau), with none in the sampled areas of the Belaga District (i.e., Lusung Laku, Sungai Koyan, and Sungai Asap).

Malaria prevalence also showed significant associations with occupation (P = 0.003) and household income (P = 0.001). By occupation, the highest proportion of cases was among non-field workers (6/29; 20.69%), followed by field workers (5/67; 7.46%), housewives (7/157; 4.46%), and dependents (i.e., a tertiary student aged 21 years old) (1/90; 1.11%). Infection was more prevalent in higher-earning households (9/50; 18.0%) than low-earning households (11/305; 3.61%). No significant association was found with prior malaria history, anemia status, or education level.

## Discussion

The study examines the prevalence of *Plasmodium* infection among 376 participants in rural communities within Kapit Division, Sarawak, from March 2024 to August 2025. The overall prevalence of *Plasmodium* infection was 5.32%. *P. knowlesi* was the predominant species, which aligns with the current trends in malaria epidemiology across Malaysian Borneo. The zoonotic *P. knowlesi* malaria now accounts for most reported cases, while indigenous human malaria has become rare [5, 7, 26]. The detection of a single case of *P. falciparum* and *P. vivax* underscores that low-level transmission of these human-specific parasites persists in rural communities.

The predominance of *P. knowlesi* observed in this study is linked to Sarawak’s ecological landscape, characterized by hilly terrain, coastal plains, and mountainous interiors, which shape malaria transmission dynamics [27]. These forested areas are habitat for the macaque reservoir, the long-tailed macaque *(Macaca fascicularis*) and the pig-tailed macaque (*Macaca nemestrina*), and the *Anopheles* vector [28, 29]. Deforestation and land-use change have increased human exposure to these natural reservoirs, facilitating the spread of *P. knowlesi* infections [30, 31]. Furthermore, most home settlements in rural areas of Sarawak, in proximity to forests and their forest-dependent livelihoods, such as farming and logging, increase the risk of infection [7–9].

A notable finding of this study is the low prevalence of submicroscopic *P. knowlesi* infections, confirming the existence of a low-density reservoir in rural settings that is undetectable by routine microscopy. Previous studies in Malaysia have documented a variable range of low-density malaria prevalence, from 0.36% to 36.9% across different settings and time periods [6, 11–19]. These studies span more than a decade, during which Malaysia experienced a substantial shift in malaria epidemiology from predominantly human malaria species to zoonotic *P. knowlesi* infections. Consequently, differences in the prevalence and species composition reported across studies should be interpreted within their respective epidemiological contexts. Consistent with our findings, several studies have identified *P. knowlesi* as the predominant species among submicroscopic infections [6, 14, 19]. Nevertheless, submicroscopic infections caused by other malaria species, including *P. vivax,* continue to be reported in certain populations and settings. For example, Jiram et al. [18] identified *P. vivax* as the most common submicroscopic species in a study conducted between 2013 and 2014 among the longhouse community in Belaga, Sarawak. These observations reflect the transition of Malaysia’s malaria landscape from predominantly human malaria species to one dominated by zoonotic *P. knowlesi,* with occasional reports of *P. vivax* infections often linked to specific outbreak periods or localized transmission contexts.

Despite the low detection rate observed, submicroscopic infections pose a challenge to elimination efforts. While the epidemiological significance of submicroscopic *P. knowlesi* infections remains incompletely understood, low-density infections may represent a hidden source of parasite persistence. However, unlike human malaria species [32], the extent to which asymptomatic and submicroscopic *P. knowlesi* infections contribute to mosquito infection and onward transmission remains unclear and requires further investigation. These infections are routinely missed by standard microscopy, particularly in low-transmission settings and asymptomatic individuals. This underscores the necessity of sensitive molecular tools, such as nested PCR, in surveillance programs for active case detection in community-based screening, outbreak detection, and routine epidemiological monitoring in endemic and post-elimination settings.

This study observed a pronounced geographic disparity in infection rates, with all positive cases identified in Kapit District and none in Belaga District. This finding aligns with spatial–temporal analyses that consistently identify the Kapit district as a high-risk area for malaria in Sarawak, with transmission often clustering in the northern and central regions [5, 33]. The heterogeneity between these two rural, forested districts suggests highly localized variations in transmission intensity. Kapit's status as a persistent hotspot can be attributed to a confluence of ecological and anthropogenic factors. A key factor is the significant overlap between human activity and the reservoir host's habitat. The distribution of long-tailed macaques (*M. fascicularis*) harboring *P. knowlesi* was predominant in districts such as the Kapit Division [33]. This macaque species is known to inhabit areas near humans due to deforestation and easier access to food [31, 34]. Furthermore, the presence of competent vectors such as *Anopheles latens*, previously incriminated in Kapit, facilitates efficient transmission [35].

In contrast, the absence of cases in the Belaga District warrants careful interpretation. This finding contrasts with a prior study conducted 12 years earlier in Belaga that reported a low prevalence (1.7%) of submicroscopic *P. vivax* malaria during a local outbreak [18]. The temporal gap between our study and this study is important, as Malaysia has since achieved and sustained zero indigenous human malaria with substantial changes in malaria epidemiology [1]. While zoonotic *P. knowlesi* infections increasingly dominate the current malaria trend in Malaysia*,* the earlier study was conducted during *P. vivax* outbreak, which may have influenced detection rates. The discrepancy may also be due to our sampling, which may have occurred during a period of low seasonal transmission or missed focal transmission hotspots. Another possible reason is that genuine differences in macaque density, vector ecology, or local behavior may exist between the two districts, warranting further investigation.

Analysis of demographic data reveals a clear association between malaria infection and older age groups, with most cases occurring in individuals over 40 years old and a complete absence of cases in children. This age distribution pattern is consistent with previous findings on *P. knowlesi* [9, 12, 13] and is associated with occupational exposure. Adults are more likely to engage in high-risk, forest-based livelihoods, such as farming, logging, or hunting, thereby increasing contact with reservoir hosts and vectors [7–9]. Whereas children may typically remain within or near the longhouse, limiting exposure.

Occupation was also significantly associated with infection, although the observed pattern deviates from conventional expectations. A higher risk was observed among non-field workers, such as teachers and office staff, compared to field workers, including farmers, loggers, and laborers. Additional cases were also found among housewives and dependents, including tertiary students. These findings indicate that malaria transmission is not limited to forest-based occupational exposure but also extends to peridomestic environments. This observation aligns with growing evidence that *P. knowlesi* transmission can occur in peridomestic environments, particularly in communities near forest fringes, where human settlements overlap with vector habitats and macaque reservoirs [7, 9, 30, 36].

In settlements near forest fringes, occupational categories alone do not fully account for transmission, and incidental exposure during routine activities contributed substantially to infection risk [9]. The high incidence of cases observed among non-forest-related occupations in this study may reflect differences in behavioral exposure and vector-avoidance practices. While individuals in forest-related occupations may adopt protective measures at work, such as wearing protective clothing or using repellents, those not routinely exposed to forest environments may be less vigilant in everyday activities. Furthermore, the primary vector, *An. latens*, is exophagic and has been documented to initiate biting as early as 18:00 h in Kapit [30, 35]. This early-evening biting behavior overlaps with routine activities such as commuting home from work, social interaction among longhouse communities, childcare, and household tasks [36]. Because these vectors predominantly bite and rest outdoors, conventional indoor-based interventions offer limited protection [37]. In contrast, children are more likely to remain indoors during these times, which may partially explain their lower observed risk.

Household income also showed a paradoxically similar pattern. This study observed a higher risk among participants with household incomes > RM500 per month than among those with incomes < RM500 per month. This contrasts with previous studies that have linked lower socioeconomic status to increased malaria risk through poorer housing conditions and reduced access to preventive measures [10, 38]. However, in a rural setting, higher income does not necessarily indicate affluence; it is a proxy for better housing conditions or the ability to acquire preventive measures such as repellent. These individuals living within a longhouse environment likely share similar baseline exposure risks. While this study did not collect behavioral data to assess these factors directly, higher earners were more likely to hold formal employment, such as non-field work. They may have greater mobility, enabling them to travel more frequently between settlements or forest-fringe districts. Increased mobility has been identified as a significant risk factor for *P. knowlesi* infection [9].

While this study's findings provide valuable insights into the prevalence of malaria in rural communities in Sarawak, several limitations should be acknowledged. First, the study uses a cross-sectional design, which provides only a snapshot of the infection at a single time point. Therefore, this study cannot establish a causal relationship between low-density parasitemia and symptom development or the acquisition of partial immunity, and thus requires a longitudinal study design. This study also used convenience sampling to recruit community members. The study was conducted in a limited number of communities or longhouses within Kapit and Belaga districts, which may not be fully representative of Sarawak's entire population. The longhouses in this study were selected based on accessibility and community willingness to participate, which may have introduced selection bias. At the individual level, participation was voluntary, and individuals who were absent during sampling or declined to participate may have different exposure risks, potentially biasing prevalence estimates and risk factor analyses. Additionally, the complete absence of cases in the Belaga district in our study may be due to a lack of transmission or an inadequate sample size to detect very low-level transmission in that specific area. As reported by Jiram et al. [18], a low prevalence (1.7%) of submicroscopic malaria was observed in a large sample of 1744 respondents.

Furthermore, the relatively small sample sizes of non-field workers (n = 29) and of households with income > RM500 (n = 50) limit statistical precision and may inflate prevalence estimates. The unequal distribution between groups may increase the risk of false-positive associations and require verification in a larger, more representative population. This study examines only certain risk factors, primarily demographic and socioeconomic information. It did not examine practice-related factors that could influence infection prevalence, such as housing conditions, personal protective measures, or vector exposure. The inclusion of these variables in future studies is essential to developing a more comprehensive understanding of malaria prevalence, risk, and transmission drivers among rural communities in Sarawak. Similarly, we recognize the exclusion of pregnant women as a limitation of this study. Although this decision was made to ensure a consistent comparator group for our prevalence and hematological analyses, pregnant women constitute a significant population in malaria-endemic regions. Future *P. knowlesi* surveillance studies should be specifically designed to include pregnant women, as their exclusion creates a significant gap in understanding the true burden and clinical impact of this emerging zoonotic infection across all at-risk groups.

Additionally, while PCR provides high sensitivity for detecting sub-microscopic infections, the findings lack data on infection density to investigate the causal relationship. The detection of only two submicroscopic cases in our study may have underestimated the true burden of asymptomatic carriers. More sensitive molecular approaches, including quantitative PCR (qPCR) for parasite quantification and ultra-sensitive methods such as reverse transcription-based total nucleic acid detection, have demonstrated improved limits of detection for *P. knowlesi* and other zoonotic malaria species [39]. Additionally, this study did not incorporate serological assessments, which can provide complementary information on historical exposure and cumulative transmission intensity [40]. The integration of serological markers with molecular diagnostics would enable a more comprehensive assessment of current and past malaria exposure in low-transmission settings, thereby enhancing the discovery of hidden reservoirs.

## Conclusions

This study provides important findings that strengthen Malaysia's path toward malaria elimination, particularly in Sarawak. The findings confirm that *P. knowlesi* is the principal cause of infection, underscoring the need to integrate a One Health approach that coordinates human, animal, and environmental health to effectively manage transmission from wildlife reservoirs. Importantly, the research shows that a significant portion of infections in rural communities are submicroscopic and would be undetected by conventional diagnostics. This evidence supports investing in advanced molecular surveillance tools and incorporating serological approaches, as the combined use of molecular and serological methods can enhance the detection of hidden reservoirs and improve the assessment of transmission dynamics. Furthermore, the study identifies clear links between infection risk and specific sociodemographic factors, and transmission is not restricted to forest-based occupations but extends to peridomestic settings and is likely shaped by behavioral and environmental exposures. This enables public health authorities to design precisely tailored interventions that reduce exposure and protect those most at risk. To maintain national elimination, a targeted strategy is recommended, one that combines enhanced detection, timely treatment, focused vector control, and community-specific prevention. Implementing this integrated approach, especially in rural-endemic areas, will be key to achieving a malaria-free Malaysia.

## Data Availability

The datasets used and analysed during the current study are available from the corresponding author on reasonable request.

## Abbreviations

DNA: Deoxyribonucleic acid
EDTA: Ethylenediaminetetraacetic acid
Hb: Hemoglobin
PCR: Polymerase chain reaction
RNA: Ribonucleic acid
WHO: World Health Organization

## References

[1] World Health Organization. World malaria report 2025. Geneva: World Health Organization; 2025.

[2] World Health Organization. Global technical strategy for malaria 2016–2030, 2021 update. Geneva: World Health Organization; 2021.

[3] Li Q, Liu T, Lv K, Liao F, Wang J, Tu Y, et al. Malaria: past, present, and future. Signal Transduct Target Ther. 2025;10:188. 10.1038/s41392-025-02246-3 PMC12170910 40523953

[4] Shahari S, Bin Abdullah ML, Binti Isman Rohimly AA, Binti Ashrat N, Amir A, Atroosh WMM, et al. The prevalence of simian malaria in wild long-tailed macaques throughout Peninsular Malaysia. Sci Rep. 2024;14:6023. 10.1038/s41598-024-54981-2 PMC10933401 38472278

[5] Ooi CH, Phang WK, Kent Liew JW, Lau YL. Spatial and temporal patterns of *Plasmodium knowlesi* malaria in Sarawak from 2008 to 2017. Am J Trop Med Hyg. 2008;104:1814–9. 10.4269/ajtmh.20-1304 PMC8103491 33755585

[6] Noordin NR, Lee PY, Mohd Bukhari FD, Fong MY, Abdul Hamid MH, Jelip J, et al. Prevalence of asymptomatic and/or low-density malaria infection among high-risk groups in Peninsular Malaysia. Am J Trop Med Hyg. 2020;103:1107–10. 10.4269/ajtmh.20-0268 PMC7470583 32618263

[7] Chin AZ, Maluda MCM, Jelip J, Jeffree MSB, Culleton R, Ahmed K. Malaria elimination in Malaysia and the rising threat of *Plasmodium knowlesi*. J Physiol Anthropol. 2020;39:36. 10.1186/s40101-020-00247-5 PMC7686722 33228775

[8] Cox-Singh J, Davis TME, Lee KS, Shamsul SSG, Matusop A, Ratnam S, et al. *Plasmodium knowlesi* malaria in humans is widely distributed and potentially life-threatening. Clin Infect Dis. 2008;46:165–71. 10.1086/524888 PMC2533694 18171245

[9] Grigg MJ, Cox J, William T, Jelip J, Fornace KM, Brock PM, et al. Individual-level factors associated with the risk of acquiring human Plasmodium knowlesi malaria in Malaysia: a case-control study. Lancet Planet Health. 2017;1:e97-104. 10.1016/S2542-5196(17)30031-1 PMC5531251 28758162

[10] Dlamini N, Hsiang MS, Ntshalintshali N, Pindolia D, Allen R, Nhlabathi N, et al. Low-quality housing is associated with increased risk of malaria infection: a national population-based study from the low transmission setting of Swaziland. Open Forum Infect Dis. 2017;4:ofx071. 10.1093/ofid/ofx071 PMC5447662 28580365

[11] Abdull-Majid N, Yap NJ, Tee MZ, Er YX, Ngui R, Lim YAL. Evidence of submicroscopic malaria parasitemia, soil-transmitted helminths, and their coinfections among forest-fringed Orang Asli communities in Peninsular Malaysia. Am J Trop Med Hyg. 2025;112:1391–9. 10.4269/ajtmh.24-0718 PMC12139561 40199271

[12] Dian ND, Muhammad AB, Azman EN, Eddie NA, Azmi NI, Yee VC, et al. Evidence of submicroscopic *Plasmodium knowlesi* mono-infection in remote indigenous communities in Kelantan, Peninsular Malaysia. Am J Trop Med Hyg. 2023;109:1081–5. 10.4269/ajtmh.23-0184 PMC10622472 37748768

[13] Fornace KM, Herman LS, Abidin TR, Chua TH, Daim S, Lorenzo PJ, et al. Exposure and infection to *Plasmodium knowlesi* in case study communities in northern Sabah, Malaysia and Palawan, the Philippines. PLoS Negl Trop Dis. 2018;12:e0006432. 10.1371/journal.pntd.0006432 PMC6001952 29902171

[14] Jiram AI, Hisam S, Reuben H, Husin SZ, Roslan A, Ismail WRW. Submicroscopic evidence of the simian malaria parasite, Plasmodium knowlesi, in an Orang Asli community. Southeast Asian J Trop Med Public Health. 2016;47:591–9.

[15] Jiram AI, Hisam S, Abdul Hamid MH, Jelip J, Noordin R, Musa KI, et al. Persistent low-density infection in areas at risk of malaria reintroduction in Malaysia. PLoS ONE. 2025;20:e0329219. 10.1371/journal.pone.0329219 PMC12633942 41264599

[16] Mat Salleh NH, Abdul Rahman MF, Samsusah S, De Silva JR, Tan JH, Amir A, et al. Complications of sub-microscopic Plasmodium vivax malaria among Orang Asli in Pos Lenjang, Kuala Lipis. Trop Biomed. 2021;38:33–5. 10.47665/tb.38.1.006 33797521

[17] Naserrudin NA, Aziz EIA, Aljet E, Mangunji G, Tojo B, Jeffree MS, et al. High incidence of asymptomatic cases during an outbreak of *Plasmodium malariae* in a remote village of Malaysian Borneo. PLoS Negl Trop Dis. 2021;15:e0009450. 10.1371/journal.pntd.0009450 PMC8205135 34081725

[18] Jiram AI, Ooi CH, Rubio JM, Hisam S, Karnan G, Sukor NM, et al. Evidence of asymptomatic submicroscopic malaria in low transmission areas in Belaga district, Kapit division, Sarawak, Malaysia. Malar J. 2019;18:156. 10.1186/s12936-019-2786-y PMC6498596 31046769

[19] Siner A, Liew ST, Kadir KA, Mohamad DSA, Thomas FK, Zulkarnaen M, et al. Absence of *Plasmodium inui* and *Plasmodium cynomolgi*, but detection of *Plasmodium knowlesi* and *Plasmodium vivax* infections in asymptomatic humans in the Betong division of Sarawak, Malaysian Borneo. Malar J. 2017;16:417. 10.1186/s12936-017-2064-9 PMC5645983 29041929

[20] Ministry of Health Malaysia. Garis panduan pengurusan malaria knowlesi di Malaysia. Edisi 1. Putrajaya: Ministry of Health Malaysia; 2025.

[21] Leedy PD. Practical research: planning and design. New York: Macmillan Publishing Company; 1993.

[22] World Health Organization. Guideline on haemoglobin cutoffs to define anaemia in individuals and populations. Geneva: World Health Organization; 2024. 38530913

[23] Singh B, Bobogare A, Cox-Singh J, Snounou G, Abdullah MS, Rahman HA. A genus- and species-specific nested polymerase chain reaction malaria detection assay for epidemiologic studies. Am J Trop Med Hyg. 1999;60:687–92. 10.4269/ajtmh.1999.60.687 10348249

[24] Singh B, Sung LK, Matusop A, Radhakrishnan A, Shamsul SS, Cox-Singh J, et al. A large focus of naturally acquired *Plasmodium knowlesi* infections in human beings. Lancet. 2004;363:1017–24. 10.1016/S0140-6736(04)15836-4 15051281

[25] Lee KS, Divis PCS, Zakaria SK, Matusop A, Julin RA, Conway DJ, et al. *Plasmodium knowlesi*: reservoir hosts and tracking the emergence in humans and macaques. PLoS Pathog. 2011;7:e1002015. 10.1371/journal.ppat.1002015 PMC3072369 21490952

[26] Ooi CH, Phang WK, Liew JWK, Atroosh WM, Lau YL. Profiling the imported human malaria in Sarawak, Malaysia in 2011–2019. Trop Med Int Health. 2011;28:486–500. 10.1111/tmi.13875 37042251

[27] de Zulueta J. Malaria in Sarawak and Brunei. Bull World Health Organ. 1956;15:651–71. PMC2538283 13404442

[28] Ang JXD, Kadir KA, Mohamad DSA, Matusop A, Divis PCS, Yaman K, et al. New vectors in northern Sarawak, Malaysian Borneo, for the zoonotic malaria parasite, *Plasmodium knowlesi*. Parasites Vectors. 2020;13:472. 10.1186/s13071-020-04345-2 PMC7490903 32933567

[29] Nada-Raja T, Kadir KA, Divis PCS, Mohamad DSA, Matusop A, Singh B. *Macaca fascicularis* and *Macaca nemestrina* infected with zoonotic malaria parasites are widely distributed in Sarawak, Malaysian Borneo. Sci Rep. 2022;12:10476. 10.1038/s41598-022-14560-9 PMC9213397 35729212

[30] Fornace KM, Brock PM, Abidin TR, Grignard L, Herman LS, Chua TH, et al. Environmental risk factors and exposure to the zoonotic malaria parasite Plasmodium knowlesi across northern Sabah, Malaysia: a population-based cross-sectional survey. Lancet Planet Health. 2019;3:e179–86. 10.1016/S2542-5196(19)30045-2 PMC6484808 31029229

[31] Stark DJ, Fornace KM, Brock PM, Abidin TR, Gilhooly L, Jalius C, et al. Long-tailed macaque response to deforestation in a *Plasmodium knowlesi*-endemic area. EcoHealth. 2019;16:638–46. 10.1007/s10393-019-01403-9 PMC6910895 30927165

[32] Schneider P, Bousema JT, Gouagna LC, Otieno S, van de Vegte-Bolmer M, Omar SA, et al. Submicroscopic *Plasmodium falciparum* gametocyte densities frequently result in mosquito infection. Am J Trop Med Hyg. 2007;76:470–4. 17360869

[33] Yunos NE, Sharkawi HM, Hii KC, Hu TH, Mohamad DSA, Rosli N, et al. Spatio-temporal distribution and hotspots of *Plasmodium knowlesi* infections in Sarawak, Malaysian Borneo. Sci Rep. 2022;12:17284. 10.1038/s41598-022-21439-2 PMC9568661 36241678

[34] Davidson G, Chua TH, Cook A, Speldewinde P, Weinstein P. The role of ecological linkage mechanisms in *Plasmodium knowlesi* transmission and spread. EcoHealth. 2019;16:594–610. 10.1007/s10393-019-01395-6 30675676

[35] Tan CH, Vythilingam I, Matusop A, Chan ST, Singh B. Bionomics of *Anopheles latens* in Kapit, Sarawak, Malaysian Borneo in relation to the transmission of zoonotic simian malaria parasite *Plasmodium knowlesi*. Malar J. 2008;7:52. 10.1186/1475-2875-7-52 PMC2292735 18377652

[36] Manin BO, Ferguson HM, Vythilingam I, Fornace K, William T, Torr SJ, et al. Investigating the contribution of peri-domestic transmission to risk of zoonotic malaria infection in humans. PLoS Negl Trop Dis. 2016;10:e0005064. 10.1371/journal.pntd.0005064 PMC5065189 27741235

[37] Naserrudin NA, Lin PYP, Monroe A, Culleton R, Baumann SE, Sato S, et al. Exploring barriers to and facilitators of malaria prevention practices: a photovoice study with rural communities at risk to *Plasmodium knowlesi* malaria in Sabah, Malaysia. BMC Public Health. 2023;23:1316. 10.1186/s12889-023-16173-x PMC10332084 37430300

[38] Dapari R, Yusop MZFM, Chinnasamy D, Zakaria NI, Shoaib SMM, Edros ME. A systematic review of the factors associated with malaria infection among forest rangers. PLoS ONE. 2024;19:e0303406. 10.1371/journal.pone.0303406 PMC11095669 38748721

[39] Braima KA, Piera KA, Lubis IND, Noviyanti R, Rajahram GS, Kariodimedjo P, et al. Improved limit of detection for zoonotic *Plasmodium knowlesi* and *P. cynomolgi* surveillance using reverse transcription for total nucleic acid preserved samples or dried blood spots. PLoS Negl Trop Dis. 2025;19:e0012129. 10.1371/journal.pntd.0012129 PMC11838868 39913530

[40] Byrne I, William T, Chua TH, Patterson C, Hall T, Tan M, et al. Serological evaluation of risk factors for exposure to malaria in a pre-elimination setting in Malaysian Borneo. Sci Rep. 2023;13:12998. 10.1038/s41598-023-39670-w PMC10415323 37563178

